# Urine Test Strip Quantitative Assay with a Smartphone Camera

**DOI:** 10.1155/2024/6004970

**Published:** 2024-03-18

**Authors:** Miroslav Pohanka, Jitka Zakova

**Affiliations:** Military Faculty of Medicine, University of Defence, Trebesska 1575, Hradec Kralove 50001, Czech Republic

## Abstract

Urine test strips for urinalysis are a common diagnostic tool with minimal costs and are used in various situations including homecare and hospitalization. The coloration scaled by the naked eye is simple, but it is suitable for semiquantitative analysis only. In this paper, a colorimetric assay is developed based on a smartphone digital camera and urine test strips. Assays of pH, albumin, glucose, and lipase activity were performed as a tool for the diagnosis of aciduria, alkaluria, glycosuria, proteinuria, and leukocyturia. The RGB color channels were analyzed in the colorimetric assay, and the assay exerted good sensitivity, and all the particular diagnoses proved to be reliable. The limits of detection for glucose (0.11 mmol/L), albumin (0.15 g/L), and lipase (2.50 U/*μ*L) were low enough to cover the expected physiological concentration, and the range for pH was also satisfactory. The urine test strips with a camera as an output detector proved applicability to spiked urine samples, and the results were also well in comparison to the standard assays which confirms the practical relevance of the presented findings.

## 1. Introduction

Urine test strips are a common tool in clinical biochemistry that serves to diagnose various diseases, including renal disease, liver disease, and some metabolic disorders. The analysis of urine for diagnostical purposes is frequently called a urinalysis. Proteins in urine, erythrocytes, specific gravity (urine osmolality), nitrites, leukocyte esterase, glucose, ketones, bilirubin, and pH are the typical markers covered by standard commercial sets. Many relevant articles have widely discussed the role and importance of urinalysis for diagnostic purposes [1–5].

The results achieved using urine test strips cannot fully replace the blood analysis. Some markers analyzed in the urine have less diagnostic importance than the same or similar markers in the blood. Glucose can be mentioned as an example. While the glucose level in the blood (glycemia) gradually increases, the glucose in the urine (glycosuria) occurs only after exceeding the renal glucose threshold. Though urinalysis has some limitations compared to the analysis of blood, it also has some significant advantages. In addition to urinary catheterization, urine collection is a noninvasive process that can be easily performed by the patient itself or by any healthcare provider. Standard urine test strips are also quite inexpensive; they can be used without any laboratory equipment or elaborative sample pretreatment, and they can be performed in the same way as point-of-care tests. Typical urine test strips are manufactured as qualitative or semiquantitative colorimetric sensors; however, the exact determination of marker concentration is not possible without specialized equipment.

Small digital cameras integrated into common electronic devices, such as smartphones and wearable technologies, are not primarily intended as a tool for laboratory analysis. However, analytical applications based on color density channel measurement and other principles have gained popularity, and many applications have been established [6–9]. The use of smartphone cameras appears to be a promising idea in the analyses [10–14]. This paper focuses on the development of a colorimetric urinalysis based on standard commercial test strips with quantification of coloration by a smartphone camera. This approach represents a novel way in analytical chemistry to improve the standard colorimetric tests designed not for instrumental analysis but for scaling by the naked eye. The urinalysis was purposely chosen as a test with a practical impact. It is expected that the use of the standard urinalysis tests in combination with a smartphone camera will provide accurate and more reproducible results suitable for practical use, making the assay more competitive to the standard laboratory methods but still useable, as a point-of-care test.

## 2. Materials and Methods

### 2.1. 3D-Printed Holder

**Figure 1:**
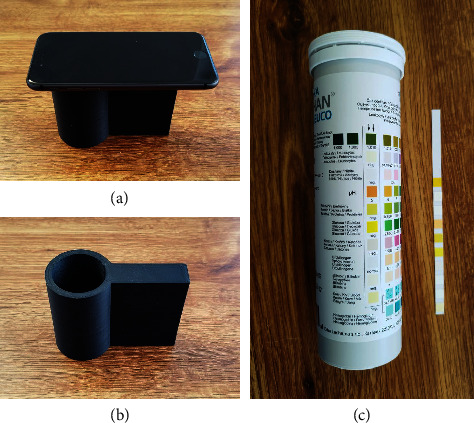
Used equipment: (a) smartphone holder with a smartphone; (b) smartphone holder; and (c) used urine test strips.

The 3D-printed holder was made from black polyethylene terephthalate glycol with a 100 mm height and an internal tube diameter of 40 mm. Prusa Mini+ (Prusa Research; Prague, Czech Republic) printer was used. The printer setting was the following: string diameter of 1.75 mm, infill of s50%, printing layer height of 0.1 mm, nozzle temperature 230°C, and bed temperature of 90°C. The holder is shown in Figure 1.

### 2.2. Reagents

Glucose, o-phenylenediamine, glucose oxidase, human serum albumin, and lipase from porcine pancreas type VI-S were purchased from Sigma-Aldrich (Saint Louis, Missouri, United States) with activity 20,000 U per protein mg (activity 1 U is equal to hydrolysis of 1.0 microequivalent of fatty acid from a triglyceride in 1 hour at pH 7.7 at 37°C using olive oil). Citric acid, sodium hydroxide, ethanol, and formaldehyde were obtained from Penta (Prague, Czech Republic). The organic and inorganic reagents used in these experiments were of analytical grade. Deionized water was prepared using the Aqua Osmotic 02 device by Aqua Osmotic, Tisnov, Czech Republic.

### 2.3. Analyzed Samples

- Sodium citrate buffer 0.1 mol/L with a pH of 5 and 6, potassium phosphate buffer 0.1 mol/L with pH 7 and 8, and sodium phosphate buffer 0.2 mol/L with pH 9.0 were used as standard samples for the pH assay. The saline served as a blank. Urine samples were used untreated.
- Glucose was solved in pH 7.4 and urine. The solutions served as samples for the glucose assay. Phosphate-buffered saline (pH 7.4) served as a blank.
- The solution of human serum albumin at pH 7.4 and the solution of human serum albumin in urine were used as standard samples for the protein assay. Phosphate-buffered saline (pH 7.4) served as a blank.
- The porcine pancreas was solved in phosphate-buffered saline (pH 7.4) and urine was used as an analyte, substituting leukocytes. Phosphate buffered saline (pH 7.4) served as a blank.
- Urine from anonymized human volunteers was used for validation purposes.

### 2.4. Urine Test Strips Assay

Standard urine test strips (DekaPhan Leuco, Erba Lachema, Brno, Czech Republic) were used for the experiment (photograph in Figure 1). A test strip contained 10 squares each to test one biochemical marker (specific gravity, leucocytes, nitrite, pH, protein, glucose, ketones, urobilinogen, bilirubin, and blood/hemoglobin). The strip was placed on a white paper surface, covered with the hollow part of the 3D-printed holder, and a smartphone (Redmi Note 11 Pro, Xiaomi Inc., Haidian District, Beijing, China) was located on the upper of the holder in a way as shown in Figure 1. After that, a sample of 10 *μ*l was applied per square and incubated for 60 seconds, respectively, 120 seconds when leukocytase activity was measured and the test strip was photographed. The smartphone camera was set to zoom 1×, automatic flash, and automatic white balance. The camera was focused on the spot where the detecting square was placed, and the picture was collected in an 8 bit jpeg format. Five different test strips were analyzed for every sample. The difference in color depth was calculated from the two photographs.

### 2.5. Measuring Color Depth

The value of color depth was measured by the software GIMP 2.10.34 (free and open-source software). The spot with the square where the analysis took place was analyzed in five randomly selected spots with a distance higher than 2 mm from the square´s edge. The color depths were measured for the red (R), green (G), and blue (B) channels. The final mean average color depth was determined. Because the 8 bit format of photographs in jpg format was acquired, the color channels can get a value between 0 and 255.

### 2.6. Standard Assays

The urine test strips and standard assays analyzed the same samples.

- The device H160 with the PHW47-SS ISFET electrode (Hach; Loveland, Colorado, United States) served to assay the pH of the samples.
- Glucose was analyzed using glucose oxidase, horseradish peroxidase, and o-phenylenediamine by a spectroscopic assay using standard 1 cm plastic disposable cuvettes and an Evolution 201 spectrophotometer (Thermo Fisher Scientific; Waltham, Massachusetts, United States) [15].
- Proteins were analyzed using the Bradford spectroscopic assay [16]. The Bradford reagent kit (Sigma-Aldrich) was chosen for the purpose, and the assay was performed in compliance with the protocol provided by the manufacturer. The assay was performed using a standard spectroscopic assay using standard 1 cm plastic disposable cuvettes and a spectrophotometer Evolution 201.
- The porcine pancreas was analyzed as a substance that mimics leukocytes and their leukocytes esterase. Indoxyl acetate served as a chromogenic substrate for an assay in standard 1 cm disposable cuvettes and the Evolution 201 spectrophotometer. Esterase activity can be easily measured by spectrophotometry using indoxyl esters [17]. In this article, the use of indoxyl acetate in the way previously described for cholinesterase and lipase assays was chosen [18–20].

### 2.7. Data Processing

In the camera-based colorimetric assay, the average color depth (five points randomly selected at a distance from the square side equal to 1/3 of the square side length) was calculated for a sample and a blank (matrix for sample solving). The difference in color depths was calculated from sample and blank assays: Δ Color depth = Color depth (blank) − Color depth (sample).

All samples were measured on a five-time repeat. The mean and standard deviation were calculated from repeated measurements. The limit of detection was determined from the calibration curves using the rule that it is equal to the point in the calibration that numerically corresponds to the triplicate of the blank assay signal (rule S/*N* = 3).

## 3. Results and Discussion

In this study, assays of four markers typical for urinalysis by a strip test were chosen. The pH assay represented an assay of an organic marker assay, the human serum albumin represented a protein marker, and the assay of lipase as a substitute for leukocytase esterase activity represented an enzymatic marker common in clinical biochemistry.

**Figure 2:**
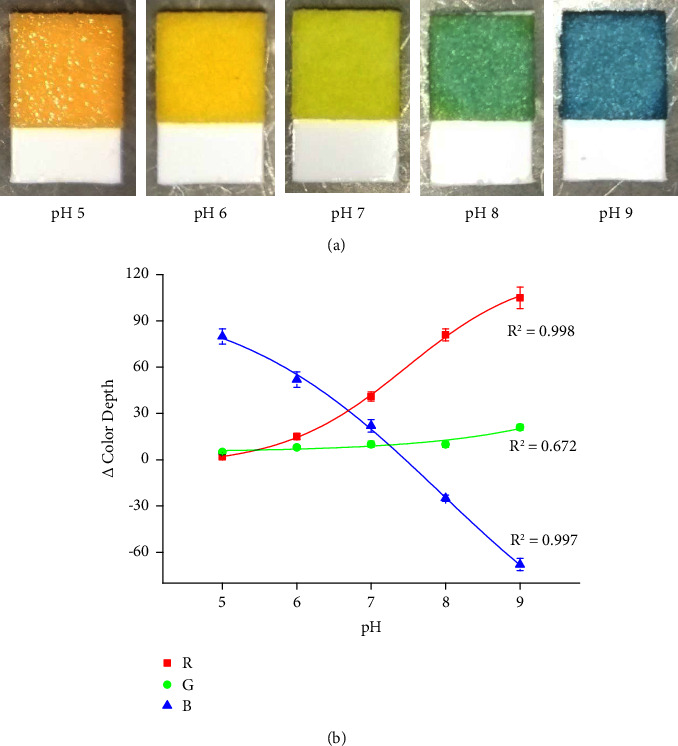
(a) Examples of urine test strips that are sensitive to pH and exposed to various buffers with pH indicated under each picture. (b) Use of urine test strips for buffers with various pH. Each sample was measured five times, and error bars indicate the standard deviation. Three curves were made for each color RGB channel.

The pH resulted in the construction of the calibration. Examples of test strip colorations and the calibration curves are shown in Figure 2. The test strips changed colors from orange in acidic buffer pH 5 per light and dark yellow (pH 6 and pH 7) to green (pH 8) and finally blue (pH 9). The sensitivity of the pH assay was very low in the G channel while the R and B channels proved to be applicable. Both R and B channels were well correlated with the change in pH, as the R channel exerted a coefficient of determination of 0.998 and the B channel had a very similar coefficient of determination of 0.997. The difference in color depth had a higher dynamic range for the B channel than for the R channel which also had a lower sensitivity for the acidic buffers. The B channel appears to be optimal for practical use, and the pH based on R and B channels is sensitive enough to cover the physiological range of urine pH and serve to recognize pathologies connected with aciduria or alkaluria.

**Figure 3:**
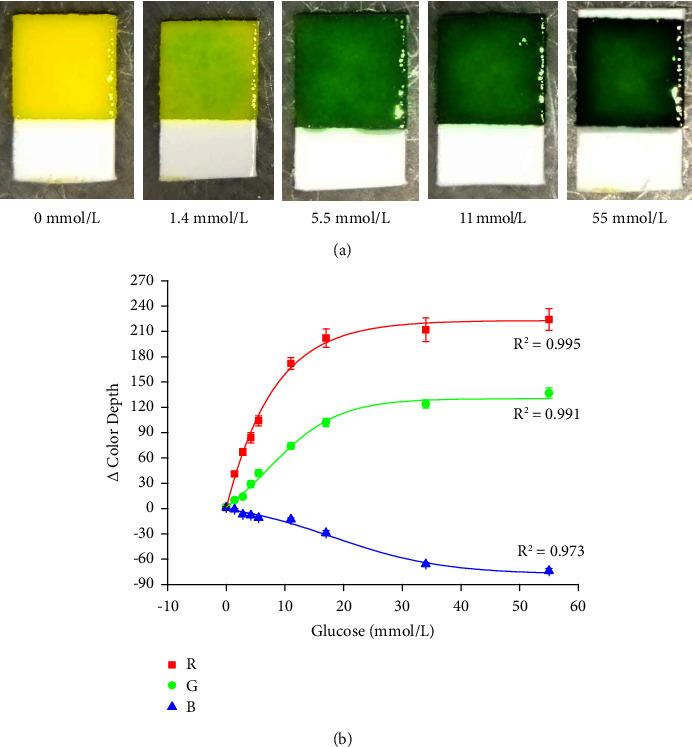
(a) Examples of urine test strips cut sensitive to glucose exposed to various glucose concentrations indicated under each picture. (b) Use of urine test strips for glucose assay. Each sample was measured five times, and error bars indicate the standard deviation. Three curves were made for each RGB color channel.

The glucose assay was performed for the calibration range of 1.4–55 mmol/L and phosphate-buffered saline served as a blank. Examples of cuts from test strips coloring in the presence of glucose and the calibration curve are depicted in Figure 3. The best sensitivity for these cuts from urine test strips was exerted by the assay in the R channel where the limit of detection of 0.11 mmol/L was reached for the glucose and the assay also had the highest dynamic range. The assay in the G channel had a lower dynamic range and limit of detection for glucose was 0.60 mmol/L. The worst result was observed in the calibration of glucose using the B channel where a limit of detection equal to 1.8 mmol/L for glucose was achieved and the dynamic range of color depths was the lowest one. The inverse proportionality of the calibration in the B channel is another notable fact. Nevertheless, calibrations in all three color channels were sensitive enough to provide limits of detection under the value of the renal threshold for glucose, which is around 10 mmol/L depending on the exact type of pathology [21, 22].

**Figure 4:**
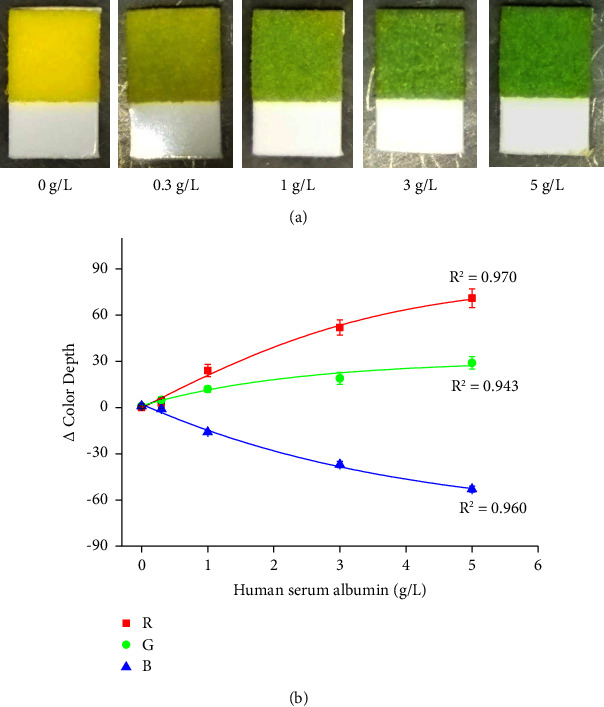
(a) Examples of urine test strips cut sensitive to proteins exposed to various human serum albumin concentrations indicated under each picture. (b) Use of urine test strips for a protein assay represented by measuring human serum albumin. Each sample was measured five times, and error bars indicate the standard deviation. Three curves were made for each RGB color channel.

The human serum albumin solution was analyzed for a calibration range of 0.3 – 5 g/L and phosphate-buffered saline served as a blank. The colors of the cuts from test strips as a result of the protein assays and calibration for human serum albumin are depicted in Figure 4. Limits of detection of 0.15 g/L for the R channel, 0.24 g/L for the G channel, and 0.22 g/L for the B channel were calculated from the calibration. The coloration of the zones for the proteinuria assay by the urine test strips came from yellow (no proteins) to green (maximal concentration of the analyzed protein). The color change was similar to the zones for the glycosuria assay, but while the glucose assay provided contrast color change, the assay of proteins led to a light green, and the dynamic range of the change in color depth was lower. While the change in color depth reached up to 200 for the glucose assay and R channel, the assay of proteinuria reached around 70 in the best R channel. The other channels exerted even lower sensitivity.

**Figure 5:**
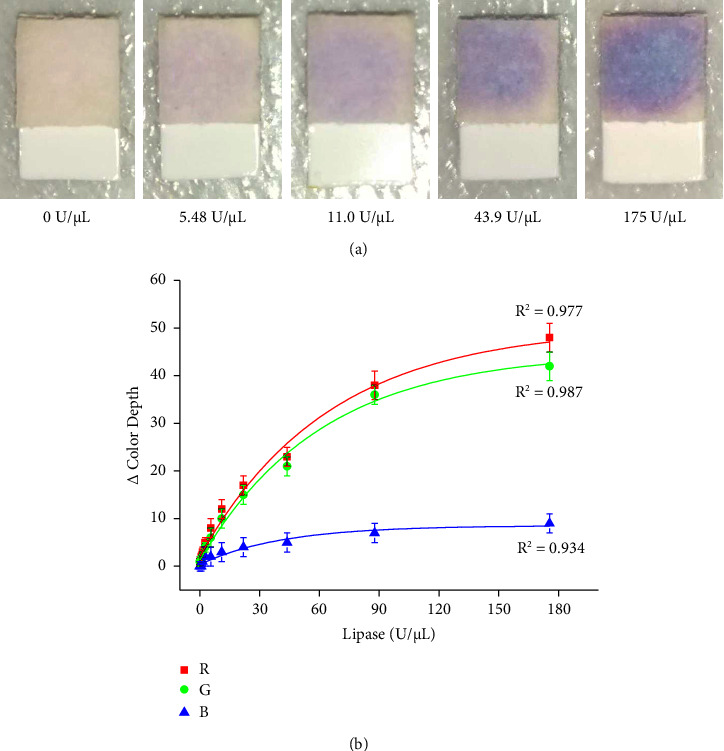
(a) Examples of urine test strips cut for leukocyte assay exposed to various activities of porcine lipase are indicated under each picture. (b) Use of urine test strips for leukocyte assay, represented by measurement of porcine lipase as a surrogate. Each sample was measured five times, and error bars indicate the standard deviation. Three curves were made for each RGB color channel.

Porcine lipase which served as a surrogate for leukocytase esterase is not a common commercially well accessible and the test for leukocyturia was verified this way. Examples of the assay and calibration of the urine test strips for lipase are presented in Figure 5. The part of the urine test strip for leukocyte detection provided quite a weak color change from colorless to a bright violet. The weak coloration resulted in a low dynamic range of the change in color depth. The best response to the color change was observed in the R channel, which was equal to approximately 50; the G channel had the maximal color change around 45; and the B channel around 10. The limit of detection for the lipase assay was equal to 2.50 U/*μ*L for the R channel, 3.40 U/*μ*L for the G channel, and 24.2. for the B channel. The activity of lipase cannot be easily recalculated to the number of lymphocytes, but an activity of 50 U/*μ*L respectively, 500 U per a standard sample 10 *μ*L is roughly equal to 500 lymphocytes considering the color etalon provided by the test strip manufacturer.

The aforementioned assays were validated using intact urine samples (pH measurement) or spiked (glucose added in the glucose assay, human serum albumin added in the protein assay, and porcine lipase added in leukocyte assays). The B channel for the pH assay and the R channels for the remaining three assays were used for the analysis of photographs from the assays by urine test strips. The exact value of the measured parameter was determined from the calibrations above using urine test strips or by standard assays calibrated using the same standards for the calibration. The results of the validations are summarized in Table 1. When comparing the data reached by the use of urine test strips and the potentiometry for pH and spectroscopy for glucose, human serum albumin, and lipase assay. In the comparison, there were no significant differences by ANOVA at the probability level of 0.05. The selected markers and their expected concentrations that can occur in the urine can be measured with urine test strips with a smartphone camera as a detector quite easily and provide data for diagnosis with similar accuracy as the standard methods.

The use of a combination of a smartphone camera and urine test strips, as presented here, falls within the scope of the idea of point-of-care urinalysis with a simple detector device. Instrumentation for point-of-care urinalysis tests has been extensively reviewed [23–26]. Several biosensor applications for particular markers in urine were also presented in recent journal articles [27–31].

## 4. Conclusions

Urinalysis by standard urine tests has limitations in the low reproducibility and limited quantification of the analyzed markers. The assay can, however, be improved by a colorimetric sensor that can even be a smartphone camera. Point-of-care tests based on this combination remain inexpensive and easy to perform but the subjective scaling of coloration by the human eye is replaced by the smartphone camera. Moreover, such an assay is quantitative and can provide results and support for precision diagnosis similar to standard laboratory methods. The urine test strips can be read by the naked eye as intended by the manufacturers; however, this method provides results highly dependent on individual abilities, sensitivities to distinguish color, and experience with such tests. The combination of smartphone and urine test strips reduces the mentioned drawbacks without raising additional costs, requiring sample processing, or introducing an elaborate assay procedure. The fact that a cheap smartphone camera integrated even into a common device is sufficient for the analysis makes the whole assay more competitive. The necessity to buy even a cheap analytical device can be a substantially limiting factor for the distribution of a point-of-care test. Because the assay needs only a common smartphone without any functions above standards, access to the improved method here is unlimited.

## Figures and Tables

**Table 1:** Validation of urine test strips with the digital camera as an output sensor for standard assays.

Marker	Urine test strip	Standard assay	Type of standard assay	Significant difference by ANOVA at *p* 0.05
pH	6.36 ± 0.19	6.41 ± 0.21	Potentiometry	No
Glucose (mmol/L)	10.6 ± 0.7	10.3 ± 0.5	Spectroscopy	No
Human serum albumin (g/L)	2.61 ± 0.31	2.56 ± 0.23	Spectroscopy	No
Leukocyturia surrogated by lipase activity (U/*μ*L)	53.7 ± 4.3	56.2 ± 3.9	Spectroscopy	No

## Data Availability

All data are included in the manuscript.
